# First Report of Cylindrospermopsin Production by Two Cyanobacteria (*Dolichospermum mendotae* and *Chrysosporum ovalisporum*) in Lake Iznik, Turkey

**DOI:** 10.3390/toxins6113173

**Published:** 2014-11-18

**Authors:** Reyhan Akcaalan, Latife Köker, Ayça Oğuz, Lisa Spoof, Jussi Meriluoto, Meriç Albay

**Affiliations:** 1 Department of Freshwater Biology, Faculty of Fisheries, Istanbul University, Ordu Cad. No. 200, Laleli, Istanbul 34470, Turkey; E-Mails: latifekoker@gmail.com (L.K.); aycaoguz@gmail.com (A.O.); albay.hermano@gmail.com (M.A.); 2 Department of Biosciences, Åbo Akademi University, Tykistökatu 6A, 20520 Turku, Finland; E-Mails: lisa.spoof@abo.fi (L.S.); jussi.meriluoto@abo.fi (J.M.)

**Keywords:** LC-MS/MS, cyanobacteria, Lake Iznik, cylindrospermopsin, Turkey

## Abstract

Cylindrospermopsin (CYN) is a cytotoxic alkaloid produced by cyanobacteria. The distribution of this toxin is expanding around the world and the number of cyanobacteria species producing this toxin is also increasing. CYN was detected for the first time in Turkey during the summer months of 2013. The responsible species were identified as *Dolichospermum* (*Anabaena*) *mendotae* and *Chrysosporum* (*Aphanizomenon*) *ovalisporum*. The *D. mendotae* increased in May, however, *C. ovalisporum* formed a prolonged bloom in August. CYN concentrations were measured by LC-MS/MS and ranged from 0.12 µg·mg^−1^to 4.92 µg·mg^−1^ as dry weight, respectively. Both species were the only cyanobacteria actively growing and CYN production was attributed solely to these species. Despite CYN production by *C. ovalisporum* being a well-known phenomenon, to our knowledge, this is the first report of CYN found in *D. mendotae* bloom.

## 1. Introduction

Toxins produced by cyanobacteria are increasingly documented around the world and problems related to toxins in water bodies have received more attention in recent years. As a result, awareness of risk posed by cyanotoxins increased substantially and regulatory approaches for cyanotoxin risk management have been put in place. Apart from a few countries (Australia, New Zealand and Brazil), microcystin is considered as a toxin for regulation or guidance value. However, Cylindrospermopsin (CYN) is increasingly encountered worldwide, including all continents with the exception of Antarctica [1].

Cylindrospermopsin was originally identified and implicated as the causative agent of the Palm Island mystery disease in 1979 [2] and its structure elucidated by Ohtani *et al.* [3]. It is a biologically active alkaloid with hepatotoxic, cytotoxic and neurotoxic effects [4]. Recent studies showed that CYN has also genotoxic effects on hepatocytic and enterocytic model cell lines of the human [5] and even lymphocyte cells [6].

Although it is named after *Cylindrospermopsis raciborski* which originates from tropical lakes of Australia and Africa*,* there are other cyanobacterial species (*Anabaena bergii*, *Anabaena lapponica*, *Aphanizomenon gracile*, *Aphanizomenon ovalisporum*, *A. flos-aquae*, *Lyngbya wollei*, *Raphidiopsis curvata*, *R. mediterranea* and *Umezakia natans*), known to produce cylindrospermopsin [7]. *C. raciborskii* is considered an invasive species because of its dispersal capacity around the world, especially into temperate climates, and successful populations have been established in various areas in recent years. On the other hand, the blooms of *C. raciborskii* generally appear to be non-toxic in Europe [8]. Another important cylindrospermopsin producer is *Chrysosporum* (*Aphanizomenon) ovalisporum*. This species was first described by Forti [9] from Lake Küçükçekmece, Turkey. However, it has not been recorded in this lake after that. In contrast to *C. raciborskii*, all populations of *C. ovalisporum* produce cylindrospermopsin. Toxic populations forming blooms have been found in Israel [10], Greece [11], Spain [12], Italy [7], and also in Australia [13]. CYN production is also attributed to other *Aphanizomenon* and *Anabaena* species with some uncertainty. Brient *et al.* [14] detected CYN in four waterbodies dominated by either *Aphanizomenon flos-aquae* or *Anabaena planktonica*. Cylindrospermopsin was also found in two German lakes without clear evidence of a known producer [15].

It is important to detect the responsible species for cyanotoxin production, especially for risk management. This study describes the presence of CYN in *Dolichospermum* (*Anabaena*) *mendotae* bloom for the first time in the world and contributes to the increasing prevalence and distribution of CYN, since it is the first report of CYN from Turkey.

## 2. Results and Discussion

Lake Iznik is a deep, alkaline lake with a high conductivity. Recorded water temperatures were greater than 20 °C when the cyanobacteria bloom samples were collected and reaching 27.4 °C in August. Water transparency was high in May and a dramatic decrease was observed in August reflected in the chlorophyll*-a* values (Table 1). Nutrient values refer to meso-eutrophic character with a high chlorophyll-*a*, TP values and low transparency. SRP was higher in late spring; however, it decreased to under detection limits by the end of August when the *C. ovalisporum* bloom was over.

**Table 1:** Some physicochemical characteristics of Lake Iznik taken on sampling days.

Parameters	Units	Date		
		22 May 2013	22 August 2013	28 August 2013
Water Temperature	°C	20.6	27.0	27.4
pH	-	8.9	8.6	9.2
Conductivity	µS·cm^−1^	900	1037	1056
Secchi depth	m	7.9	1.4	1.4
Nitrate + Nitrite	µg·L^−1^	125.6	391.4	69.6
Soluble Reactive Phosphorus	µg·L^−1^	11.3	2.5	<2
Total Phosphorus	µg·L^−1^	24.8	12.7	13.4
Total Nitrogen	µg·L^−1^	1889	1562	1253
Chlorophyll-*a*	µg·L^−1^	4.2	20.9	23.1

The genus *Dolichospermum* is distinguished from *Anabaena* with some characteristics including the obligatory presence of gas vesicles, intercalary and frequently solitary heterocysts, undifferentiated apical cells and solitary or small clusters of filaments [16].

**Figure 1:**
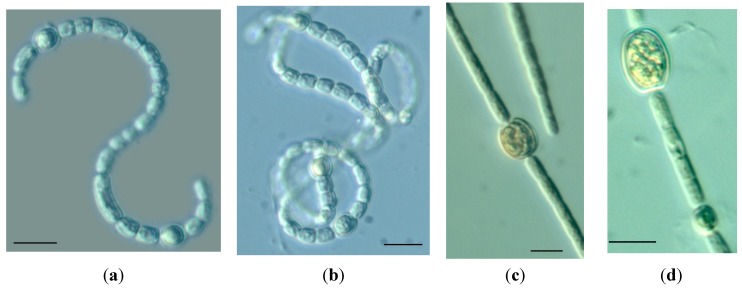
Trichomes of *Dolichospermum mendotae* (**a**,**b**) and *Chrysosporum ovalisporum* (**c**,**d**) showing vegetative cells, heterocysts and akinets. Scale bars indicate 10 µm.

The trichomes of *Dolichospermum* from Lake Iznik are free floating, loosely coiled, solitary or sometimes in small clusters. Vegetative cells are cylindrical and there is no difference in the apical cell. Heterocysts are oval, slightly wider than vegetative cells. They are found solitary and intercalary in the trichome. Akinets are intercalary, solitary and elongated with rounded ends (Figure 1). The morphometric characteristics of the vegetative cells, heterocysts and akinetes of *Dolichospermum* are given in Table 2.

The 16S rRNA gene regions were also amplified from *Dolichospermum* bloom sample from Lake Iznik and BLAST search was done to confirm the species. 16S rRNA gene sequence showed 99% identity to previously published 16S rRNA gene sequence of *D. mendotae* strain CHAB 4408 and CHAB 3512.

**Table 2:** The size and the length/width ratio of vegetative cells, akinetes and heterocysts of *D. mendotae* and *C. ovalisporum*.

Species	Morphological characters	Form	Mean ± SD	Min	Max	*n*
***D. mendotae***						
	Cell					
		length	4.8 ± 0.7	3.4	5.8	50
		width	4.2 ± 0.3	3.5	5	
		l:w	1.1	0.98	1.2	
	Heterocyst					
		length	6.5 ± 0.8	4.7	8.4	50
		width	5.6 ± 0.5	4.1	6.6	
		l:w	1.2	1.15	1.3	
	Akinete					
		length	8.96 ± 1.8	7.2	11.3	5
		width	4.8 ± 0.7	4.1	5.7	
		l:w	1.9	1.8	2	
***C. ovalisporum***						
	Cell					
		length	6.1 ± 1.8	2.9	13.4	50
		width	3.4 ± 0.4	2.7	4.4	
		l:w	1.8	1.1	3	
	Heterocyst					
		length	6.2 ± 0.7	4.9	7.9	50
		width	4.3 ± 0.7	2.7	6.5	
		l:w	1.4	1.8	1.2	
	Akinete					
		length	12.1 ± 2	7.9	15.5	15
		width	9.6 ± 1.8	6.9	14.5	
		l:w	1.3	1.1	1.07	

The species was identified as *Dolichospermum mendotae*. The dimensions of vegetative cells, akinetes and heterocysts are smaller in comparison to the literature. On the other hand, there is a direct link between the cell dimensions and nutrient availability. The phosphorus concentrations have a clear effect on cell dimensions. Zapomelova *et al.* [17] found that the cell length and length/width ratio decreased at higher phosphorus concentrations. Morphologically, the dimensions of the cells and the length/width ratio are important characteristics to identify the species. This situation was discussed on the identification of *D. mendotae* and *D. sigmoideum*, since the only morphological difference between them is the width and the length/width ratio of vegetative cells and cell wall constriction [18]. Therefore, these are considered as a joint morphological group. On the other hand, Zapomelova *et al.* [17] indicated that the cell dimensions of these two species are only slightly different and spanned in the range of both species in culture conditions. Therefore, we decided to define the species as *D. mendotae* instead of using the term of *D. mendotae/sigmoideum* complex, since it possesses a priority over *D. sigmoideum* according to Botanical code [17]. Moreover, 16S rRNA results confirmed the species as *D. mendotae*.

*D. mendotae* is a part of phytoplankton community of meso to eutrophic lakes [19]. The distribution area is very wide from the Southern to Northern hemisphere and has been detected in more than ten countries around the world (Table 3). Although it was recorded in different continents, generally these records are based on strains in culture collections or floristic studies in the water bodies and there is very little information about its biomass in environmental samples. The blooms are reported from Lednice ponds, Czech Republic [20], Lake Karhijarvi in Finland [21], and in Lake Stechlin [22].

**Table 3:** The examples of geographical distribution and toxicity of *D. mendotae* and *C. ovalisporum*.

Species	Status	Geographical Area	Toxicity	Reference
***D. mendotae***	Environmental	Finland	NA	[21]
		Bangladesh	NA	[23]
		Brazil	NA	[24]
		Hungary	NA	[22]
		Germany	NA	[25]
		Poland	NA	[26]
		Czech Republic	NA	[20]
		Greece	NA	[27]
	Strain	Finland	Ana	[28]
		Japan	NA	[29]
		Denmark	NT	[30]
		Spain	NT*	[31]
***C. ovalisporum***	Environmental	Israel	CYN	[10]
		Greece	MCY	[11]
		Spain	CYN	[12]
		Italy	CYN	[7]
	Strain	Israel	CYN	[10]
		Australia	CYN	[13]
		USA	CYN	[32]
		Spain	CYN	[31]

NA: Not Analyzed; NT: No Anatoxin, Microcystin; NT*: No Anatoxin, Microcystin, Cylindrospermopsin and PSP; Ana: Anatoxin-a; CYN: Cylindrospermopsin; MCY: Microcystin.

*Chrysosporum* (*Aphanizomenon*) *ovalisporum* was identified in accordance with morphological features [19]. *C. ovalisporum* has straight, solitary, free-floating filaments. Vegetative cells are barrel shaped and are longer than wide (Table 2). Apical cell is rarely hyaline. Heterocysts are elipsoidal and placed in the middle of trichome and connected to the neighboring cell with a small bridge. Akinetes are oval to nearly spherical and distant from each other (Figure 1).

According to 16S rRNA analysis, the similarity between published sequences and the environmental sample in this study was 100% for *C. ovalisporum*. The identification of *C. ovalisporum* was therefore confirmed by both morphologic and molecular methods.

*C. ovalisporum*, now considered to be an invasive species [8] in Europe, was first detected in Lake Küçükçekmece located in the Istanbul metropolitan area in 1910 by Forti and dominated the phytoplankton community in Lake Kinneret in 1994. During the last decades, its distribution area expanded from Greece to the Iberian Peninsula (Table 3). Up to now, there has been no bloom record in Turkish freshwaters; however, this could be a result of insufficient monitoring efforts. This species is not restricted by special environmental conditions. It can be found in both deep, stratified waterbodies and shallow ponds [8]. The bloom in Lake Iznik was observed at the beginning of August when the water temperature was over 25 °C and conductivity was 1034 µS·cm^−1^. Temperature is an important driving factor for *C. ovalisporum* growth as well as dispersion. The bloom in different reservoirs took place at a temperature over 25 °C [11,12,33].

A total of three bloom samples were analyzed for cylindrospermopsin (Table 4). CYN was analyzed from one sample taken in May 2013 which contained *D. mendotae* and 2 samples collected in August 2013 when *C. ovalisporum* formed a bloom. *D. mendotae* was collected on a GF/C filters (Whatman, Maidstone, UK), whereas filaments of *C. ovalisporum* were collected with a plankton net and then separated from other species using its buoyancy and subsequently freeze-dried. Both species are the only cyanobacteria found in the bloom samples. A 100 µm length of *C. ovalisporum* filament was regarded as one unit, however, cell counts was done for *D. mendotae*,since it has tangled filaments. *D. mendotae* formed stripes on the surface in the morning of a calm day at Lake Iznik. On the other hand, *C. ovalisporum* reached higher numbers producing a heavy bloom as also indicated in biomass results. Moreover, it lasted for a longer period, nearly a month.

**Table 4:** The abundance and biomass of *D. mendotae* and *C. ovalisporum* and cylindrospermopsin (CYN) concentrations.

Species and toxin	Units		Date		
			22 May 2013	22 August 2013	28 August 2013
***D. mendotae***	Abundance	Cell·L^−1^	4.9 × 10^7^	-	-
	Biomass	µg·L^−1^	3471	-	-
***C. ovalisporum***	Abundance	Fil·L^−1^	-	2.3 × 10^7^	2.0 × 10^7^
	Biomass	µg·L^−1^	-	20,960	18,413
**Cylindrospermopsin**		µg·L^−1^	0.12	3.91	4.92

**Figure 2:**
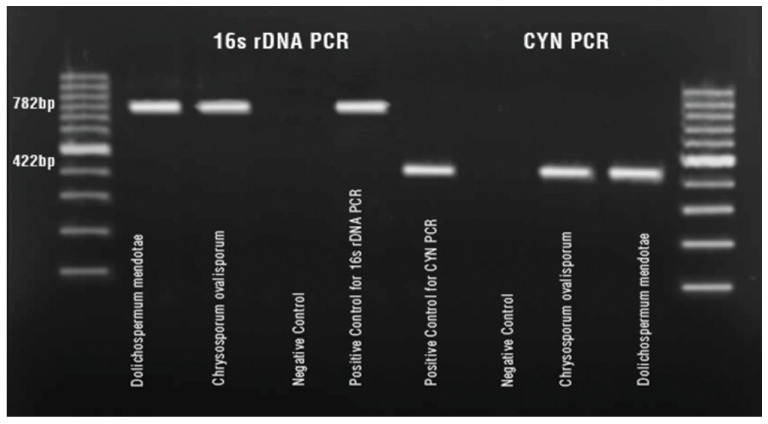
PCR assay for 16SrRNA and polyketide synthase genes of CYN in bloom samples.

*D. mendotae* and *C. ovalisporum* bloom samples were investigated for the presence of polyketide synthase (*cyrC* and *aoaC*) genes of the CYN cluster. A PCR product at 422 bp was obtained in bothbloom samples showing the potential of CYN production of these two species (Figure 2) [34].

**Figure 3:**
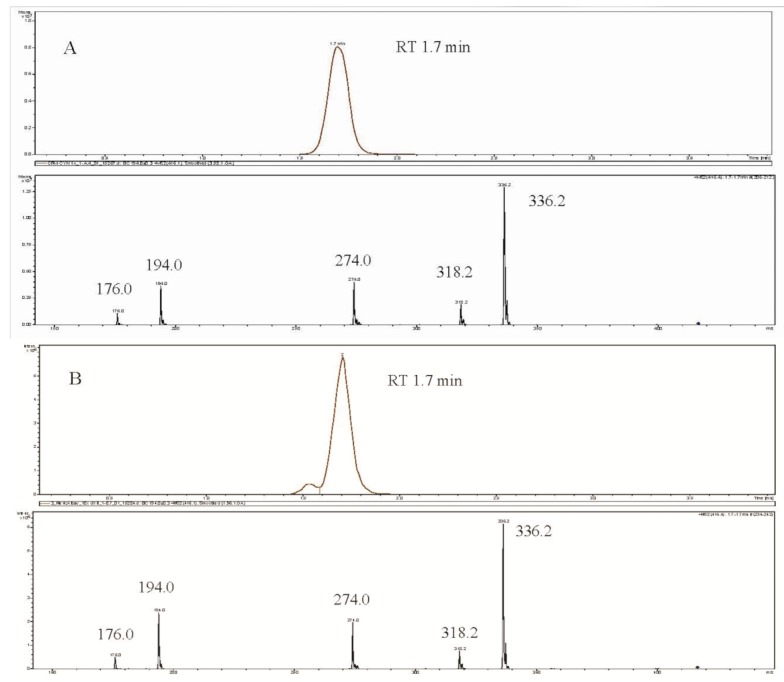

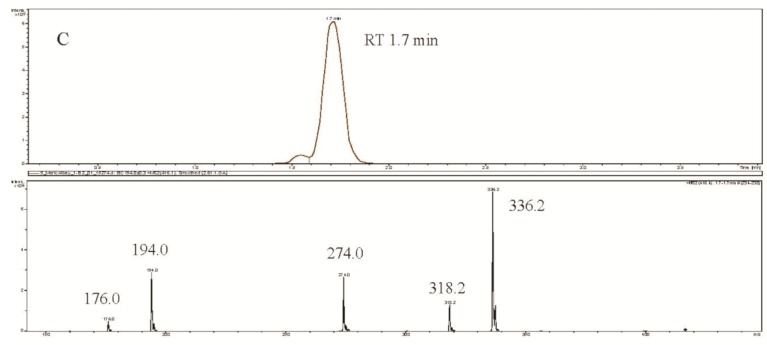
Extracted Ion Chromatogram (EIC) of *m*/*z* 416 > 194 (Upper panel) and MS/MS spectrum of *m*/*z* 416 (Lower panel) of CYN standart (**A**), *C. ovalisporum* (**B**), *D. mendotae* (**C**).

CYN production was also detected using LC-MS/MS based on extracted ion chromatograms and MS/MS spectra of CYN standards and samples. The retention time of samples matched that of the standard, 1.7 min (Figure 3).

This is the first report of CYN production of *D. mendotae* bloom. It is confirmed both the presence of polyketide synthase genes and LC-MS/MS. The only toxin reported to be produced by *D. mendotae* in the literature was anatoxin-a (ATX-a) [29]. The Ana producing strain was isolated from Lake Säyhteenjärvi located in the Southern part of Finland. Cires *et al.* [32] screened the cyanotoxin production of Nostocalean strains including *D. mendotae* from Spain and could not detect any MC, ATX, CYN and PSP toxin production or responsible genes in their strain. Similarly, MC and ATX production were not detected in *D. mendotae* strain 57 isolated from Lake Velje Sø, Denmark [31]. The lack of information of toxin production by this species might be due to the bloom formation patterns. This species generally produce blooms at the beginning of the growth season, in May, as it is observed in Lake Iznik and the bloom formation is generally very weak. The stripes can be found on the surface of water when it is very calm and disappear easily with a light wind. This makes it difficult to detect the bloom. 

*C. ovalisporum* became the main CYN producer in Southern Europe. Our results showed that the *C. ovalisporum* bloom in Lake Iznik had higher CYN concentration compared to the reported concentrations from other countries [7,12]. Moreover, the detected concentrations fall within the upper range of maximum CYN concentrations found in isolated strains of different CYN producers [32]. On the other hand, the concentration of CYN detected in the *D. mendotae* bloom is markedly lower (0.12 mg·g^−1^) in comparison to *C. ovalisporum*. As only intracellular CYN was detected in *D. mendotae* sample, the result could be an underestimate. The bloom period coincided with the growth season of Cladocera species in Lake Iznik and their gut contents were full of cyanobacteria. Therefore, it is necessary to confirm the extra- and intracellular CYN production with isolated strains of *D. mendotae*. Moreover, the effect of grazers on the changes of intra- and extracellular CYN concentrations also needs to be clarified.

## 3. Experimental Section

### 3.1. Study Site

Lake Iznik is a natural lake located in the western part of Turkey. It is the fifth biggest lake of Turkey with a surface area of 308 km^2^ and maximum depth of 65 m. Partially treated sewage and non-point pollution of fertilizers and pesticides from surrounding agricultural areas reach the lake [35]. Lake water is also used for irrigation and is a popular place for recreation during summer months. 

### 3.2. Sample Collection

Samples were collected from surface waters when there was a surface bloom of *Dolichospermum* (*Anabaena*) *mendotae* in May 2013 and another bloom of *Chrysosporum* (*Aphanizomenon*) *ovalisporum* in August 2013. The bloom of *C. ovalisporum* persisted nearly one month and samples were collected twice with one week intervals.

Samples for microscopic observation were immediately fixed with 1% Lugol’s solution. Water samples for nutrient and chlorophyll-*a* analysis were stored in cold and dark conditions and transported to the laboratory. Temperature, conductivity, dissolved oxygen and pH was measured by portable multiparameter (Sonde Model 6600, YSI Incorporated, Yellow Springs, OH, USA) and Secchii disc measurements were done with a 20 cm black and white device.

Cyanobacterial species were enumerated according to Utermöhl [36] and 100 µm length of *C. ovalisporum* filament was regarded as one unit. However, since *D. mendotae* had tangled filaments, the measurement of filament lengths was not possible; therefore, cell counts were performed. After the measurement of the dimensions biovolumes were calculated using the formulae of Hillebrand *et al.* [37]. Chlorophyll-a measurements were performed according to Nusch [38]. Nutrient analysis was done according to APHA, AWWA and WEF [39].

### 3.3. Molecular Analysis

Total genomic DNA extraction from lyophilized samples was performed using XS extraction buffer containing 1% potassium-methylxanthogenate; 800 mM ammonium acetate; 20 mM EDTA; 1% SDS; 100 mM Tris-HCI (pH 7.4) [40].

16s rDNA amplification was performed using forward primer (27F; 5' AGA GTT TGA TCC TGG CTC AG 3') and reverse primer (809R; 5' GCT TCG GCA CGG CTC GGG TCG ATA 3'). Thermal cycling was performed at 92 °C for 2 min followed by 35 cycles of 94 °C for 10 s, 60 °C for 20 s and 72 °C for 1 min and a final extension step at 72 °C for 5 min [41].

Amplified PCR products of 16S rDNA were visualized on 1.5% agarose gels stained with ethidium bromide and photographed under UV transillumination. Published sequences were obtained from NCBI databases (http://www.ncbi.nlm.nih.gov). Genbank accession numbers of the sequences obtained in this study are KM360484 for *D. mendotae* and KM360485 for *C. ovalisporum*.

CYN polyketide synthase (*aoa*C and *cyr*C) genes of CYN gene cluster were amplified using forward primer (K18; 5' CCTCGCACATAGCCATTTGC 3') and reverse primer (M4; 5' GAAGCTCTGGAATCCGGTAA 3'). Thermal cycling conditions for the PCR were 94 °C for 10 min; 94 °C for 30 s, 45 °C for 30 s, and 72 °C for 30 cycles; 72 °C for 7 min [34]. PCR products were visualized on agarose gel and DNA molecular weight marker was used to indicate the size of the amplification products. *Cylindrospermopsis raciborskii* AWT 205 was used as a positive control.

### 3.4. CYN Extraction

Water sample was filtered through a GF/C filter in May 2013 when *D. mendotae* increased in high numbers and *C. ovalisporum* was separated from water by using its buoyancy in August 2013. Samples were lyophilized and extracted with 100% methanol containing 0.1% TFA and the extracts dried with nitrogen. The dry extracts were dissolved in 300 μL of water and filtered through GHP Acrodisc 13 mm syringe filters (Pall Life Sciences, Ann Arbor, MI, USA) with 0.2 µm GHP membrane. Then samples were analyzed for cylindrospermopsin using LC-MS/MS as described below.

### 3.5. CYN Analysis Using the LC-MS/MS

The LC-MS/MS experiments were carried out on an Agilent Technologies (Waldbronn, Germany) 1200 Rapid Resolution (RR) LC coupled to a Bruker Daltonics HCT Ultra Ion trap MS (Bremen, Germany) with electrospray (ESI) source. The 1200 RR LC system included a binary pump, vacuum degasser, SL autosampler, and thermostated column compartment set at 40 °C. Separation of the toxins was achieved on a Supelco (Bellefonte, PA, USA) Ascentis C_18_ column, (50 mm × 3 mm I.D. with 3 µm particles) protected by a 4 × 2 mm C_8_ guard column. The mobile phase consisted of solvents A, 99% water–1% acetonitrile–0.1% formic acid; B, acetonitrile–0.1% formic acid with the following linear gradient program: 0 min 0% B, 2.5 min 0% B, 2.6 min 50% B, 4 min 50% B, 4.1 min 0% B; stop time 10 min. The flow-rate was 0.5 mL·min^−1^ and the injection volume was 5 µL.

The ion trap was operated utilizing positive electrospray ion mode. The ion source parameters were set as follows: evaporator temperature 350 °C, nebulizer pressure 40 psi and dry gas flow 10.0 L·min^−1^. The capillary voltage was set at 4.0 kV. The MS scan range was *m*/*z* 395 to 440 and MS/MS fragmentation of the target mass *m*/*z* 416 was employed to get MS/MS spectra. The ICC target was set to 200,000 with a maximum accumulation time of 100 ms.

CYN in the samples was identified by comparing the retention time (1.7 min) and MS/MS fragmentation with those of the pure CYN standard (12.5 µM CRM-CYN, NRC-IMB, Halifax, NS, Canada). 1:10, 1:50, 1:100, 1:200 and 1:500 aqueous dilutions of the standard were analyzed for quantification purposes.

## 4. Conclusions

In conclusion, reports on the CYN distribution around the world have been expanding due to an increasing awareness of cyanotoxin problems. CYN became the second most studied freshwater toxin after microcystin and pose more hazards to water users since the liberation of CYN to surrounding water is much higher compared to other toxins. Therefore, the identification of CYN producers is of great importance and the determination of CYN in the bloom of *D. mendotae* was revealed for the first time with this study. Moreover, this is the first report of CYN in Turkish freshwaters. In terms of water management, there are very limited national regulations or guideline values considering CYN in contrast to microcystin [42]. On the other hand, the production of CYN by two different cyanobacteria species growing in the same water body in different time of growth season increase the risk to exposure to the toxin. Therefore, to identify the producers and also to detect the CYN concentrations from different regions will be helpful to manage and prevent potential health hazards to people. 
